# Interaction of Age, Discrimination, and Vigilance on Memory-Concentration Difficulty in a National U.S. Sample

**DOI:** 10.1093/geroni/igaf122.2525

**Published:** 2025-12-31

**Authors:** Roger Wong, Emily Norris

**Affiliations:** SUNY Upstate Medical University, Syracuse, New York, United States; SUNY Upstate Medical University, Syracuse, New York, United States

## Abstract

Discrimination and vigilance have been linked to adverse physical and mental health conditions. There is no existing research that has examined how the intersections between age, discrimination, and vigilance may influence cognition. This study examines whether the frequency of discrimination and vigilance are associated with memory-concentration difficulty, and whether these relationships vary by age group. The 2023 National Health Interview Survey administered the five-item Everyday Discrimination Scale (e.g. receive poor customer service) and four-item Heightened Vigilance Scale (e.g. avoid social situations) for a nationally representative sample of 28,583 U.S. adults, who were categorized into four age groups: young adult (18-24), early middle-age (25-39), late middle-age (40-64), and older adult (65+). Average scores (range 0-3) were computed separately from the discrimination and vigilance scales. After applying survey sampling weights and adjusting for sociodemographic, geographic, and health covariates, the logistic regressions indicated that multiple monthly instances of discrimination and vigilance were associated with an increased odds of memory-concentration difficulty by 2.7 times (OR = 2.72, 95% CI = 2.06-3.62, p<.001) and 3.0 times (OR = 2.98, 95% CI = 2.58-3.45, p<.001) times, respectively, compared to those with no exposure. There was also a significant age-discrimination (p=.02) and age-vigilance (p <.001) interaction on memory-concentration difficulty. The highest probabilities of memory-concentration difficulty were among older adults with the most frequent exposure to discrimination and vigilance behaviors. These results suggest that discrimination and vigilance may be a factor contributing to the heightened risk for cognitive impairment among older adults. Future research is needed to examine the underlying mechanisms behind these age-related differences in memory-concentration.

